# Farm to Work: Development of a Modified Community-Supported Agriculture Model at Worksites, 2007–2012

**DOI:** 10.5888/pcd12.150022

**Published:** 2015-10-22

**Authors:** Christina A. Thi, Karissa D. Horton, Jennifer Loyo, Esbelle M. Jowers, Lindsay Faith Rodgers, Andrew W. Smiley, Eric Leversen, Deanna M. Hoelscher

**Affiliations:** Author Affiliations: Karissa D. Horton, Jennifer Loyo, Limetree Research, LLC, Austin, Texas; Esbelle M. Jowers, The University of Texas at Austin, Texas; Lindsay Faith Rodgers, Texas Department of State Health Services, Austin, Texas; Andrew W. Smiley, Sustainable Food Center, Austin, Texas; Eric Leversen, WebChronic Consulting, LLC, Austin, Texas; Deanna M. Hoelscher, The University of Texas Health Science Center at Houston, School of Public Health, Austin, Texas.

## Abstract

**Background:**

The Farm to Work program is a modified community-supported agriculture model at worksites in Texas.

**Community Context:**

The objective of the Farm to Work program is to increase fruit and vegetable intake among employees and their households by decreasing cost, improving convenience, and increasing access while also creating a new market for local farmers at worksites. The objectives of this article were to describe the development, implementation, and outcome of a 5-year participation trend analysis and to describe the community relationships that were formed to enable the successful implementation of the program.

**Methods:**

The Farm to Work program began in November 2007 as a collaborative effort between the nonprofit Sustainable Food Center, the Texas Department of State Health Services, the Web development company WebChronic Consulting LLC, and Naegelin Farm. The program provides a weekly or biweekly opportunity for employees to order a basket of produce online to be delivered to the worksite by a local farmer. A 5-year participation trend analysis, including seasonal variation and sales trends, was conducted using sales data from November 2007 through December 2012.

**Outcome:**

The total number of baskets delivered from November 2007 through December 2012 was 38,343; of these, 37,466 were sold and 877 were complimentary. The total value of sold and complimentary baskets was $851,035 and $21,925, respectively. Participation in the program increased over time and was highest in 2012.

**Interpretation:**

The Farm to Work program increased access to locally grown fruits and vegetables for employees and created a new market for farmers. Increased program participation indicates that Farm to Work can increase employees’ fruit and vegetable consumption and thus help prevent chronic diseases in this population

## Background

Consumption of fruits and vegetables in the United States is lower than recommended, and several government health goals and initiatives focus on increasing it (1–4). A diet high in fruits and vegetables helps prevent chronic diseases and may decrease obesity prevalence (5,6). Chronic diseases are the main cause of death in the United States, and 34.9% of US adults and 17% of youth are obese (2,5,7,8).

An effective way to increase fruit and vegetable consumption is through community-supported agriculture (CSA) programs. These programs consist of individuals who purchase farm shares, thereby distributing the farm’s risks and benefits, and regularly receive a portion of crops during the growing season (9). There are more than 6,900 CSAs in the United States, and 200 are in Texas (10). Among low-income families who participate in CSAs, household fruit and vegetable variety and inventories increase, but consumption does not (11).

The Farm to Work program is a modified CSA model designed to increase fruit and vegetable consumption by providing access to fresh produce at worksites. This program does not require participants to purchase a share but instead provides a weekly or biweekly opportunity to order a basket of produce online to be delivered to the worksite by a local farmer. The basket consists of 12 to 15 pounds of fresh produce, including mostly vegetables and some fruits and herbs; a trade basket is available for participants to trade produce items. This modified CSA model supports local farmers and has the added convenience of delivery to the worksite. The objectives of this article are to describe the development and implementation of the Farm to Work program, to outline community engagement efforts and community relationships that were formed, and to present outcomes of a 5-year participation trend analysis.

## Community Context

Similar to national consumption, consumption of fruits and vegetables is low in Texas; in 2011, the median reported daily consumption rate among Texas adults was 1.0 time per day for vegetables and 1.6 times per day for fruits (1). The median daily consumption rate among Texas adolescents aged 14 to 18 years was 1.0 time per day for vegetables and 1.0 time per day for fruits (1). Barriers to fruit and vegetable consumption, such as high cost, perceived lack of time, and limited access, are well-documented in a diverse, multiethnic, general US population and in a multiethnic worksite population (12,13). The objectives of the Farm to Work program are to prevent diet-related chronic diseases by addressing Healthy People and US Dietary Guidelines goals of increasing fruit and vegetable consumption; to decrease the cost of, improve the convenience of, and increase access to fruits and vegetables for employees and their households; and to create viable sales outlets for local farmers at worksites.

Development of the Farm to Work program required new partnerships that would be mutually beneficial to all organizations. The community engagement effort created a relationship between worksites and farmers. Outcomes of interest for this partnership were increased fruit and vegetable consumption among employees and their households, development of a new market for farmers, relationship formation between farmers and consumers through worksites, increased engagement of consumers and farmers in the local food system, increased access to fruits and vegetables from small farms, and increased knowledge among Farm to Work participants of the local food system related to the seasonality and source of local produce.

## Methods

The Farm to Work program began in November 2007 as a collaborative effort between the nonprofit Sustainable Food Center (SFC), the Texas Department of State Health Services (DSHS), the Web development company WebChronic Consulting LLC (WebChronic), and Naegelin Farm. SFC, a nonprofit organization with a mission to “cultivate a healthy community by strengthening the local food system and improving access to nutritious, affordable food,” operates in central Texas and was identified as a potential partner because of its established relationships and reputation with local farmers and its work in the local food system, including other “farm to institution” endeavors (14). DSHS was charged with implementing policy, systems, and environmental changes to increase the consumption of fruits and vegetables through an obesity prevention cooperative agreement with the Centers for Disease Control and Prevention (CDC). In response, DSHS engaged SFC and WebChronic to formalize the concept. DSHS designed and provided marketing materials and ensured that Farm to Work could legally operate at state agencies. The DSHS main campus was the first worksite to participate in the Farm to Work program, which enabled pilot testing and further refinement of the protocols. WebChronic had previously worked with DSHS to create an online reporting system and had the requisite skills to create an online purchasing and administrative system to organize and store data related to ordering and email distribution at each worksite. Worksites were identified as partners, because they were a new market segment that did not already participate in existing markets of CSAs and farmers markets.

In developing a method to distribute produce, the partners first considered conventional models, such as CSAs and farmers markets, to increase the availability and intake of locally grown fruits and vegetables at worksites. In most CSAs, members must purchase a basket at consistent intervals (usually weekly or biweekly) for an entire growing season. CSAs do not allow flexibility for times when the member is unable to pick up or use the produce. The standard CSA model was modified by replacing the weekly or biweekly commitment with a weekly or biweekly opportunity to order a basket, which was made possible by the collective purchasing power at worksites. Farm to Work was intended to reach employees who did not already engage in the local food system and needed to overcome a cost and convenience barrier. The program also provided an additional way of purchasing local food to those employees who already participated in the local food system, such as those who had developed relationships with CSAs and farmers markets. As a state agency worksite, DSHS represented a mixture of employees with various levels of socioeconomic status. This program reached the entire worksite, providing flexibility for the employees and an additional source of income for the farmer.

The partners collaboratively implemented the program. A coordinator employed at each worksite volunteered to oversee internal operations, including produce delivery and pickup, program promotion, and direct customer service for program participants. Volunteer coordinators were usually self-selected; they spent a minimum of 2 hours per delivery week on distributing produce, providing customer service, and promoting the program. SFC initially ran the production side of Farm to Work operations by recruiting and training farmers, matching worksites to farmers, and providing ongoing technical support to farmers. The DSHS Nutrition, Physical Activity, and Obesity Prevention Program developed a protocol to implement Farm to Work through worksites and operated the consumer side of Farm to Work by training and providing ongoing support to coordinators. Over time, the responsibility of training and providing ongoing support to coordinators transferred to SFC.

SFC contracted with WebChronic to create and maintain the online system. To date, the cost of developing the online order site and administrative system, including initial development and upgrades, is approximately $29,000, and 3% of the purchase price covers monthly maintenance costs. Each worksite had a unique Web address for their ordering website, and the data were linked to an administrative website.

The Farm to Work program was launched in November 2007 at the DSHS main campus in Austin and expanded to its second worksite, Austin State Hospital, in December 2007. The program launch was funded by an obesity prevention grant from CDC, which was used to organize a communications plan and purchase promotional materials. The DSHS Communications Unit developed a logo and artwork for the program. DSHS, SFC, and WebChronic created a promotional video and placed it on the Farm to Work website to encourage employees to participate. Communications and promotional materials were developed for dissemination through other worksites to promote the program. For example, logo and artwork files were sent to coordinators when the program was launched at their worksite. These worksites then personalized the marketing materials to meet their own needs. Initially, volunteer coordinators also received a complimentary basket, but this practice was discontinued in the second year of the program due to rules at some worksites related to receiving gifts.

Partner communication was essential to promotion of the program. Partners communicated with one another via email and in-person meetings. Farm to Work was promoted to employees through wellness fairs, posters, emails, and a link placed on organizations’ wellness websites. DSHS and SFC met with interested worksite staff to manage expectations related to the content of the basket, online system, and responsibilities. Communication to the public was achieved through the Farm to Work website and the Farm to Work webpage on the SFC and DSHS sites.

The Farm to Work program was evaluated using mixed methods in 2007, 2008, 2010, and 2011 (Table). All evaluation instruments were custom created for the program, and other evaluations may have been done at other worksites.

**Table:** Farm to Work Program Evaluation Methods, Texas Worksites, 2007–2012

Timeframe	Evaluation Method/Respondent Type (n)	Constructs (No. of Questions)	Administration
June 2007	Survey to inform program development; employees from DSHS Main, Austin State Hospital, DSHS Exchange, DSHS Howard Ln (n = unknown)	Fruit and vegetable content (n = 6) Source of meals (n = 3) Behaviors related to fruit and vegetable consumption (n = 5) Worksite environment (n = 3) Food buying and preparation (n = 4)	Online survey was emailed to 3,527 employees
December 6, 2007	Intercept interview; participants from DSHS Main (n = 19)	Program participation (n = 2) Attitudes toward local foods (n = 1) Produce consumption (n = 1) Food preparation (n = 1)	In person at produce distribution
December 6, 2007	Intercept interview; DSHS Main participants (n = 19)	Program participation (n = 2) Logistics (n = 2)	In person at produce distribution
December 6, 2007	Intercept interview; DSHS Main participants (n = 20)	Program participation (n = 3)	
2007	Survey; farmer (n = 1)	Amount of land farmed (n = 2) Sales and income (n = 2) Labor costs (n = 2) Travel related to delivering produce (n = 2) Challenges, benefits, changes related to staffing, work hours, equipment and supplies, and other feedback (n = 4)	Unknown
2008	Survey; farmer (n = 1)		Unknown
2010	Focus group informed employee survey; coordinators (n = unknown)	Ways to increase participation	In person
2010	Survey; participants from all 19 worksites (n = 707)	Product quality, quantity, variety Process Outreach and promotion Information Website usefulness Employee behavior (n = 49 total)	Online survey link was placed on emailed order reminders, order receipts, pick-up reminders, and on worksite wellness and intranet web page
2010	2 Focus groups; frequent participants 2 Focus groups; nonparticipants and infrequent participants (n = 27 total)	Employee knowledge and interaction with farmer website information Program incentives	In person; participants were recruited with a flyer and received a $20 stipend and a free dinner
2010	Interview; farmers (n = 4)	Participation satisfaction related to the product, process, outreach and promotion, information provided, website usefulness, fruit and vegetable consumption, cooking practices	In person
Spring 2011	Survey; DSHS Main employees (n = 373)	Fruit and vegetable content (n = 6) Source of meals (n = 3) Behaviors related to fruit and vegetable consumption (n = 5) Worksite environment (n = 3) Food buying and preparation (n = 4)	Online to approximately 2,000 employees

Abbreviation: DSHS, Texas Department of State Health Services.

Participant and partner feedback were used to further refine the program to increase satisfaction for participants and farmers. Results from participant satisfaction surveys conducted in 2010 informed best practices for farmers, which included providing high-quality, fresh produce, adding variety to increase value, ensuring accurate information about the produce list, having a trade box regardless of the number of customers, using creative marketing strategies to inform participants, and making recommendations to improve the website. Results from the 2010 participant satisfaction survey informed the start of a promotion that rewarded participants with a complimentary basket for every 10 baskets ordered during a 1-year period. Participant concerns with the volume and variety of produce offered were addressed by farmers rotating produce in the baskets, providing a trade basket, and ensuring at least 12 to 15 pounds of produce per basket. In 2012, WebChronic contracted with a website usability expert who gathered insights from SFC, DSHS, worksite coordinators, and participants. The findings from the usability testing and participant satisfaction surveys conducted in 2010 informed updates to the ordering and administrative websites in spring 2013. Website features added as a response to user needs included a recipe section and an encyclopedia of produce called the Vegipedia. The interface and design of both websites supported ease of use and consistency in program branding. Employees could ask questions and provide feedback at distribution, use the feedback feature on the order website, or respond to order reminder emails; online questions and feedback were routed to the respective worksite coordinator to reply to the employee.

Success of community engagement efforts was assessed with process-related data collected through the online system. The ability to track participation by the number of baskets and sales by worksite was built into the Farm to Work administrative site. SFC, WebChronic, and DSHS used the data for quarterly reports, invoices, and evaluation of participation and sales, respectively. SAS 9.2 (SAS Institute, Inc) was used to analyze the participation and sales data. Analysis of variance (ANOVA) was used to determine if there was a significant difference in participation by season and by month. Because there were significant differences in participation by season and by month, a Student-Newman-Keuls post-hoc test was used to determine which seasons and months were statistically different from the others.

## Outcomes

In 2013, a summative mixed-methods evaluation of the program was conducted. A 5-year participation trend analysis, including seasonal variation and sales trends, was conducted using sales data from November 2007 through December 2012. This included trends in the number of baskets delivered and participating worksites (Figure). Participation increased and showed seasonal variations over the 5-year period. Participation was evaluated as the number of delivered baskets; the number of participants could not be assessed, because participants may share baskets and the online ordering and administration systems do not save participant information to protect privacy. A monthly time series participation analysis (Figure) indicated a linear trend in participation that significantly increased every 4 months (*t* = 2.96, *P* = .004) and every 7 months (*t* = 2.57, *P* = .01). The trend line increased by a slope of 2.947 baskets per month. However, participation decreased from 2009 to 2010 (Figure). Participation at each site tended to decrease from year to year, and the increase in participation from the 5 new sites in 2010 did not offset the decrease in participation in worksites that continued from 2009. The limited variety and quality of produce that resulted from drought may have also affected the overall decrease in participation from 2009 to 2010.

**Figure:**
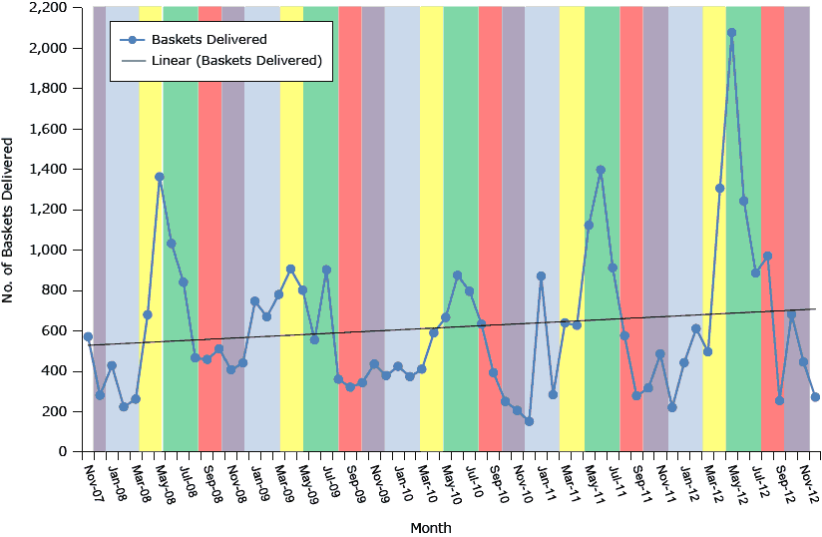
Trends in total number of baskets delivered by month, Texas Worksites, November 2007 through December 2012. Shading denotes seasons. **Table:** 

Month	No. of Baskets Delivered						
	2007	2008	2009	2010	2011	2012	Total
January	0	428	746	424	871	442	2,911
February	0	223	670	373	284	611	2,161
March	0	262	780	410	639	496	2,587
April	0	679	906	590	627	1,306	4,108
May	0	1,363	801	666	1,123	2,077	6,030
June	0	1,033	555	875	1,397	1,244	5,104
July	0	841	903	796	913	886	4,339
August	0	466	360	633	575	971	3,005
September	0	458	321	393	278	254	1,704
October	0	511	343	250	317	682	2,103
November	571	407	435	206	485	446	2,550
December	281	441	378	151	220	271	1,742
Total	852	7,112	7,198	5,767	7,729	9,686	38,344

From November 2007 through December 2012, the total number of baskets delivered was 38,343; of these, 37,466 were sold and 877 were complimentary. Over the 5-year period, the total value of sold baskets was $851,035, and the total value of complimentary baskets was $21,925. Price per basket differed by worksite and changed over time, ranging from $10 (2 baskets) to $20 (17,122 baskets) and $25 (20,343 baskets). Participation in the program was highest in 2012, which was likely due to continued growth in the number of participating worksites. A 1-way repeated measures ANOVA indicated that a significantly higher number of basket deliveries occurred in early summer (May, June, and July) compared with the other growing seasons (*F*[9,17] = 32.74, *P* < .001); however, results of the Student–Newman–Keuls post-hoc test indicated there was no significant difference when comparing the other 4 growing seasons (late summer, fall, winter, spring). Basket deliveries were highest in early summer (mean = 2,984.6) and lowest in fall (October and November; mean = 752.5). Average basket sales for the remaining 3 growing seasons ranged from 896.8 (late summer: August and September) to 1,302.6 (spring: March and April).

Worksite participation also increased over the 5-year period. Farm to Work was implemented in 22 government worksites, 9 private industry worksites, 6 nonprofit worksites, and 4 education worksites. The number of participating worksites increased every year over the 5-year period, with 41 unique worksites; of these, 35 participated in 2012. Worksites with the highest participation across all 5 years were: The City of Austin, the DSHS main campus, the Texas Comptroller of Public Accounts, the DSHS Austin State Hospital, the Texas Capitol, and the Texas Education Agency. These worksites may also represent the largest employee populations. Of the 41 worksites, 31 had at least one person who received a complimentary basket. Five sites stopped participating during their first year because their participation was too low to justify the farmers’ time and effort; most of these sites had fewer than 50 employees. Other than these 5 sites, every site continued to participate in the program each year; some breaks occurred due to seasonal availability and disasters on the farms. In 2012, 9 farmers participated in the program.

Several barriers were overcome to launch the program. These barriers included legal concerns, the uncertainty of a new business venture, and lack of knowledge related to the local food system. A Farm to Work program champion at DSHS worked for 1 year to ensure that Farm to Work complied with policies at government worksites, building and facility requirements, vendor-related policies, or state, city, or municipal codes (15). For example, a waiver of liability was created for the farmer to sign that limited the delivered items to raw, unprepared produce. This ensured that Farm to Work did not compete with visually impaired food service vendors operating on state, federal, and other property through opportunities provided by the Business Enterprises of Texas Program, which operates under the federal authority of the Randolph-Sheppard Act. DSHS also scheduled produce delivery after the cafeteria closed for the day. The Farm to Work Toolkit developed by DSHS in partnership with SFC and WebChronic provided information, tools, sample documents, and other resources to implement the Farm to Work program at DSHS, as well as more information about addressing legal concerns (www.dshs.state.tx.us/CWWObesityF2W/) (16).

To build trust with the first farmer to commit to a new program, SFC recruited Naegelin Farm, which had communicated a need for help in increasing sales. SFC trained farmers on how to use the administrative site and on the production needs of the program. On the consumer side, the support from the worksite itself and worksite coordinators created the trust necessary for employees to purchase the produce. DSHS and SFC trained coordinators on how to order a basket, what their involvement entailed, and expectations related to basket contents. To address participant perceptions of the produce being more expensive than grocery store produce, coordinators conducted price comparisons with prices of comparable produce from conventional grocery stores and shared findings with participants.

Unexpected successes included the amount of profit generated for the farmers and a waitlist of worksites. There also has been both state and national recognition and interest in replicating the program at other sites. Farm to Work was recognized as an innovative program by national organizations such as CDC’s Center for Training and Research Translation, Association of State and Territorial Health Officials, and the National Association of County and City Health Officials.

## Interpretation

The Farm to Work program created a new access point for locally grown fruits and vegetables and created a new market for farmers at worksites. The program expanded to 35 active worksites in 2012 and generated $851,035 in sales over a 5-year period from November 2007 through December 2012. Other communities interested in setting up a Farm to Work program can download the Farm to Work Toolkit or consider attending SFC’s Program Replication training to receive hands-on training and a program replication guide (16,17). The time for a new worksite to start the Farm to Work program ranges from 3 weeks to 8 or more weeks, assuming that a farmer is available to supply the site. The lead time includes time to determine interest, obtain signatures on agreements, arrange logistics, conduct initial outreach, and launch. To access the online system, new communities work with WebChronic to license the software.

The Farm to Work program has increased access to locally grown fruits and vegetables for consumers and created a new market for local farmers; increasing participation levels in the program suggest that it has the potential to increase fruit and vegetable consumption and prevent diet-related chronic diseases.
